# Diet in Disease

**Published:** 1893-08-05

**Authors:** Ernest Hart

**Affiliations:** Bachelier-ès-Sciences-es-Lettres (rèstreint), formerly Student of the Faculty of Medicine of Paris, and of the London School of Medicine for Women


### Diet in Disease.

By Mrs. Ernest Hart, Bachelier-ès-Sciences-es-Lettres (rêstreint), formerly Student of the Faculty of Medicine of Paris, and of the London School of Medicine for Women.

### XXVII.—ULCER OF THE STOMACH.

When dyspepsia is of long duration and severe in character, and is accompanied with persistent vomiting and acute pain after food, then ulcer of the stomach may be suspected; if to these symptoms is added the vomiting of blood, then there is little room for doubt. Ulcer of the stomach is a most intractable disease, and its situation in a hollow organ which is subject to incessant movement during the process of gastric digestion, renders it very difficult to treat.

Ulcer of the stomach is of different kinds; it may be small, circular, or oval in shape, with sharp, perpendicular edges, and looking as if a portion of the mucous membrane of the stomach had been punched out; or it may be large and spreading, with thickened sloping edges. The floor of the ulcer may be formed of the outer coats of the stomach; or it may be constituted by one of the adjoining organs, the liver or pancreas, which has become adherent to the stomach by the process of inflammation and ulceration having extended to its surface. Sometimes gastric ulcers cicatrise and heal, sometimes they remain quiescent for a time, giving the false impression that cure has taken place, but the ulcerative process often begins again, and in many cases they end in perforation. Perforation may take place into the peritoneum, into the colon or intestine, or into an artery, in which case profuse hæmorrhage occurs. Sometimes, however, ulceration and infiltration of the tissues may extend to the adjoining organs, and a communication may even thus be established between the stomach and the external air through the abdominal walls.

**The Symptoms of Gastric Ulcer** are pain, vomiting, and hæmatemesis or bleeding from the stomach. The pain is characteristic. It occurs almost immediately after taking food, and it is either felt at the epigastrium, which becomes tender on pressure, or it is referred to the region of the spine corresponding to the last two or three dorsal or first two or three lumbar vertebræ, or to the region between the shoulders, the muscles on either side often being tender; or again it may occupy the umbilicus or some area or point near, and when severe it radiates from its chief point of intensity towards the œsophagus, backwards to the loins, or downwards and laterally over the whole of the abdomen. The pain, when severe, is of a burning, boring, and shooting character, attended with a sense of soreness. Vomiting is a later symptom, but is generally very persistent. The pain and vomiting occur soon after the ingestion of food. Hæmorrhage occurs from time to time, the bleeding being from the excoriated surface of the ulcer, or when very profuse from the erosion of an artery.

The tendency of gastric ulcer is towards recovery; and when death occurs it is from perforation of an artery causing profuse hæmorrhage, or from perforation into the peritoneum, when collapse and death take place in a few hours, or from perforation into the viscera. Death may be caused by exhaustion in consequence of the patient sinking under the long continued pain and vomiting, and becoming worn out by the want of food. (Bristowe).

It is obvious that in this distressing and painful malady diet is of the greatest importance, the necessity and aim being to maintain the strength of the patient by such food as can be digested without provoking movements in the stomach, and without causing the dreaded pain and vomiting. The object must be to give the stomach as much rest as possible, so that cicatrisation of the ulcer can go on uninterruptedly. Food must therefore be given in very small quantities at a time, and at short intervals. In cases where there has been severe hæmorrhages, and where it is likely to recur, the stomach must be kept absolutely at rest, and the patient fed by nutrient enemata for a few days at least.

**An Exclusive Milk Diet** is, as a rule, the diet indicated, and this is well borne in ulcer of the stomach. The casein of milk is thrown down as curds on coming in contact with the gastric juice of the stomach, and these curds are often difficult of digestion, especially the curds of cow's milk, which are large and flocculent. To prevent their formation the milk should be mixed with an equal quantity of lime water or an alkaline water. Dr. Burney Yeo recommends that to every four ounces of milk should be added ten grains of bicarbonate of sodium, five grains of light magnesia, ten grains of common salt, and a tablespoonful or two of water. This may be taken every two or three hours. The yolk of an egg beaten up with two tablespoonfuls of hot water may, if required, be added to the cup of milk, and given twice a day, or an ounce of the crumb of a stale roll, well soaked previously in ho water, may be mixed with the milk two or three times a day. (Yeo.) Buttermilk is recommended by some physicians, as curdling of the milk in the stomach is thus avoided, but the sour taste of the buttermilk is disliked greatly by some patients. Malt Extract is recommended by some physicians, and a *purée* of potatoes in cases where a vegetable food is well borne.

In some cases a milk diet cannot be endured—there is a distinct intolerance of milk—and recourse must be had to bouillons and *purées* of meat. The meat or chicken must be reduced to a fine pulp and mixed with a little broth or beef tea. Leube's soluble meat is much used in Germany in these cases; it is said to be so prepared that it is ready for immediate absorption without the action of the gastric juice, and if this is the fact, it must be a valuable preparation for ulcer of the stomach, where it is necessary to obtain healing by rest.

Hot tea and coffee, gruel, porridge, and alcoholic drinks are strictly forbidden. After two or three weeks of this restricted diet, and if the symptoms of pain and vomiting have disappeared, there may be a gradual return to more solid food, but the greatest caution must be exercised not to take more food than is absolutely required for the support of the body.

### Useful Recipes for Convalescents.

Purée of Chicken.—Remove all the skin and bones from part of a roast chicken. Chop the meat, pound it in a mortar, and rub it through a sieve. Take the bones of the chicken and boil them for several hours with a shallot, a small piece of carrot, two leaves of celery, a bouquet of herbs, and enough water to cover them. Strain through a hair sieve and remove all the fat. Add the pounded meat, and simmer until it is sufficiently thick; then add half a gill of cream, a few drops of lemon juice, and a small lump of sugar.

Creme de Volaille.—Melt half an ounce of butter and half an ounce of flour together in a saucepan, and add half a gill of white stock. Take the flesh of half a chicken, chop, pound it, and rub it through a sieve. When the mince is cool, add one egg and half a pint of whipped cream, and mix all together. Put it into a buttered mould and steam for a quarter of an hour.

Steamed Sole.—Skin and fillet a sole; wash and dry the fillets, and put them in a jam-pot just large enough to hold them; sprinkle a little salt and lemon juice over them, and cover them with a buttered paper. Put the jam-pot into a saucepan half full of boiling water. Cover it tightly, and let it boil for ten minutes. Mix an ounce of butter with one ounce of flour in a saucepan over the fire, add one gill of milk and liquor from the fish, and cook for ten minutes, stirring well. Pour this sauce over the fillets, and garnish with slices of lemon and a sprig of parsley on the top of each fillet.*

* "Art of Feeding the Invalid." Published at the Scientific Press (Limited), 428, Strand, W.C.

